# Protective Effects of *Clinacanthus nutans* (Burm.f.) Lindau Aqueous Extract on HBV Mouse Model by Modulating Gut Microbiota and Liver Metabolomics

**DOI:** 10.1155/2023/5625222

**Published:** 2023-01-03

**Authors:** Rui Zhang, Zhe Lu, Yachen Xu, Qian Peng, Man Xiao, Shenggang Sang

**Affiliations:** ^1^Department of Clinical Laboratory, Hainan Women and Children′s Medical Center, Haikou 570206, China; ^2^School of Tropical Medicine, Hainan Medical University, Haikou 571199, China; ^3^Department of Biochemistry and Molecular Biology, Hainan Medical University, Haikou 571199, China; ^4^Department of Clinical Laboratory, The First Affiliated Hospital of Hainan Medical University, Haikou 570102, China

## Abstract

**Background:**

*Clinacanthus nutans* (Burm.f.) Lindau (*C. nutans*) has been used in the therapy of hepatitis B (HB) and is effective; however, the mechanism of action has not been elucidated.

**Objective:**

To investigate the protective effects of *C. nutans* aqueous extract on the hepatitis B virus (HBV) mouse model based on correlation analysis between gut microbiota and liver metabolomics.

**Materials and Methods:**

We firstly constructed the animal model by high-pressure injection of pcDNA3.1(+)/HBV plasmid into the tail vein and treated it with *C. nutans*. The biomarkers and inflammatory cytokines of HB were detected by enzyme-linked immunosorbent assay and quantitative PCR; the Illumina-MiSeq platform was used for investigating gut microbiota; the LC-MS/MS method was utilized on screening liver tissue metabolites; multiomics joint analysis was performed using the R program.

**Results:**

Compared with the modeling group, *C. nutans* significantly decreased the expression levels of HBsAg, IL-1*β*, TNF-*α*(*P* < 0.05) in the serum, and cccDNA (*P* < 0.05) in the liver tissues of mice. *C. nutans* dramatically reduced the ratio of Firmicutes and Bacteroidetes (*P* < 0.05) and significantly declined the proportion of *Lactobacillaceae* and *Lactobacillus*(*P* < 0.05), dramatically increasing the relative abundance of *Bacteroidales_S24-7_*group, *Rikenellaceae*, and *Alistipes*(*P* < 0.05); LC-MS/MS analysis results showed that *C. nutans* dramatically upregulate hippuric acid, L-histidine, trehalose, D-threitol, and stachyose and downregulate uridine 5′-diphosphate, cholic acid, trimethylamine N-oxide, CDP-ethanolamine, and phosphorylcholine (*P* < 0.05). The correlation analysis revealed that *C. nutans* affects the related metabolite levels of hippuric acid and cholic acid through the modulation of crucial bacteria (*Alistipes*) (*P* < 0.01), exerting specific anti-inflammatory effects.

**Conclusion:**

These results suggest that *C. nutans* exerts protective effects in HBV model mice, showing the therapeutic potential for anti-HBV infection.

## 1. Introduction

HBV infection, which remains a severe public health problem [1], causes over 400 million people to be infected and 1 million patients to die each year [2]. Interferon and nucleoside analogs are used for treatment. However, these approved therapeutic agents are still not reliable [3]. Therefore, new strategies are needed to make the therapeutic effect better. Presently, the induction of the HBV mice model by using a hydrodynamically injected method to simulate the natural process of human infection could be a mature technology for new drug research of HBV [4].

*C. nutans* is a plant that belongs to the family of *Acanthaceae*, possessing potential and diverse medicinal values in tradition for snake and insect bites, skin rashes, herpes simplex virus, and varicella-zoster virus lesions [5]. The extract of *C. nutans* has various biological effects, such as anti-inflammatory, antioxidant, immune response, antimicrobial, and antidengue activity [6].

Gut microbiota has excellent significance for some physiological and pathological processes. Healthy flora has characteristics of richness, diversity, and stability of the intestinal ecosystem and can self-regulate and recover after being disturbed. Dysbiosis refers to the changes in gut microbiota driven by a series of factors, including the composition and function aspects, which exceed the resistance and resilience of microorganisms themselves [7]. Gut microbiota disorders are highly correlated with HB [8]. Research shows that gut microbiota changed significantly between HB patients and healthy ones [9]. The use of fecal microbiome transplantation can promote the HBeAg clearance rate of patients after persistent antiviral therapy [10]. In mouse models, an imbalance of gut microbiota may damage HBV-specificT-cell response and prolong the course of the disease [11].

The interaction between gut microbiota and liver diseases has aroused widespread interest. Bile acids, one of the hepatic metabolites, affect the intestinal secretion system of microorganisms through the hilum and bile [12–14]. Sterile mice can prevent the pathogenic role of intestinal microorganisms in viral hepatitis [15]. In patients with HBV infection, the imbalance of the microbiome promotes the increase of cytokine secretion, which induces the malignant progress of chronic inflammation and liver lesions [16, 17].

Persistent hepatitis can seriously damage gut microbiota and liver metabolism function [18]. At the same time, abnormal metabolites are also highly correlated with liver diseases. High levels of aromatic amino acids in serum may be a significant marker of liver tissue dysfunction and the pathogenesis of chronic liver diseases [19–23]. Studies have found that gut microbiota regulates the host's metabolic phenotype [24, 25]. Therefore, elucidating the regulation of gut microbiota and metabolite characteristics of HB will help reveal the further mechanism of the efficacy of *C. nutans* and lay a foundation for the development of potential medicinal plants.

## 2. Materials and Methods

### 2.1. Preparation of Plant Extract

*C. nutans* was purchased from a local pharmacy (Wuzhishan Wanjiabao Technology Co., Ltd., Haikou, China) in June 2019 and had been authenticated by Dr. Qiang Liu, School of Pharmacognosy, Hainan Medical University (Haikou, China). Fifteen grams of *C. nutans* was weighed for each serving, 500 mL of double distilled water was added and boiled for 4 h, filtered it with a 0.22 *μ*m filter element, centrifuged at 8000 g, and stored at −20°C after lyophilization. The dry powder was dissolved directly by ddH_2_O to 0.2 mL for each mouse.

### 2.2. Animals

Male BALB/cJGpt mice at the age of 5∼7 weeks were purchased from Jiangsu GemPharmatech Co. Ltd. (Nanjing, China). The mice were housed at the specific pathogen-free animal center of Hainan Medical University (Haikou, China) according to the regulations of the animal care committee. They were housed by 5 animals per cage with a temperature of 23 ± 2°C and a 12 hour light/dark cycle, receiving normal feeding. This work was approved by the Ethics Committee of Hainan Medical University for animal care and use.

### 2.3. Induction of the Animal Model and Drug Administration

The plasmid pcDNA3.1(+)/HBV (provided by Dr. Xin Wang, Ocean University of China, Qingdao, China) was used in this study. 10 *μ*g of plasmid DNA was diluted with physiological saline according to 0.1 mL/g of the mouse body weight, and then the solution was injected into the tail vein of the model group mice within 10 s. Six-week-old male mice were hydrodynamically injected (HI) with plasmids [26]. The control group mice were HI with physiological saline as the same protocol. After 24 hours of HI, the serum of mice was collected; then, we used ELISA kits (Kehua Bio-engineering Co. Ltd., Shanghai, China) to detect ALT and HBsAg levels, each sample was preprocessed by diluted 1 : 10 with PBS. The modeling mice were selected and distributed into the *C. nutans* aqueous extract group (YDC), the modeling group (MOD), and the entecavir solution group (ETC), with 10 mice per group. And 10 mice of the control group were selected as the nondiseased group (NOR). All animals weigh approximately. The group of YDC and ETC mice were orally administered 0.2 mL reagent (as described in the preparation of plant extract) or 0.2 mL entecavir. We calculated the equivalent doses for humans and mice, a group of YDC mice was gavaged 1.95 g/kg/d *C. nutans* for 10 days, a group of ETC mice was gavaged 3.2 mg/kg/d entecavir, and the other group of mice was gavaged with the same volume of distilled water.

### 2.4. Measurement of Biomarkers and Inflammatory Cytokines of HB

On day 10, all animals were sacrificed, then the blood samples were collected in sterile Eppendorf tubes by the method of blood taking from the retroorbital vascular plexus of mice, and centrifugated at 2000 g for 10 mins to separate serum. After that, the liver tissue was excised on the ice and cut into two equal parts, fast stored in liquid nitrogen until use. The HBsAg, IL-1*β*, and TNF-*α* in the serum were detected in the same protocol as the preceding description. Versus ∼1 gm of the liver tissue was taken and cut into pieces, we added 300 *μ*L DNA Dentaured&Fragmented buffer with 10% protease K ( Solarbio Bio-Engineering Co., Ltd., Shanghai, China.) in each tube to resuspend the tissue homogenate. Then, incubated it at 55°C for 2 h and centrifugated for 5 mins (at 12,000 g and room temperature). After that, we added 300 *μ*L DNA extract reagent. We added 300 *μ*L isopropanol, mixed it upside down, set it at −20°C for 1 h, and centrifugated it for 5 mins again. Discarded the supernatant and added 300 *μ*L 75% ethanol, mixed it well, and centrifugated it for 5 mins. Added 50 *μ*L ddH_2_O after discarding the supernatant and stored at 4°C overnight. The next day, Plasmid-Safe™ATP-dependent DNase was used to digest DNA. Finally, the relative contents of cccDNA were determined with mouse GAPDH expression levels. Primers are as follows:

- cccDNA-Forward, 5*′*-GTGCACTTCGCTTCACCTCT-3*′*
- cccDNA-Reverse, 5*′*-AGCTTGGAGGCTTGAACAGT-3*′*
- mGAPDH-Forward, 5*′*-CATGGCCTTCCGTGTTCCTA-3*′*
- mGAPDH- Reverse, 5*′*-ATGCCTGCTTCACCACCTTCT-3*′*

### 2.5. Microbiota Analysis by 16S rRNA Gene Sequencing

The experimental site is in Beijing Allwegene Technology Co., Ltd., Beijing, China. The feces samples were collected in sterile Eppendorf tubes and kept at −80°C until analysis. We used DNA Isolation Kit (MoBio Laboratories, Carlsbad, CA) to extract DNA from feces samples and agarose gels (0.8%) to check on the purity and quality of genomic DNA. The 16S V3-4 region was amplified with the primers as reported in [27]. Pretreatment of specimens, PCR experiments, and product purification were carried out by the operation manual. After that, we used the Miseq platform to perform deep sequencing. The raw data were trimmed using Illumina analysis pipeline version 2.6. The dataset was then analyzed using QIIME. The clustering of operational taxonomic units (OTUs) for calculating richness and diversity indices is as reported in [28]. All sequences were classified by the ribosomal database project (RDP) classifier tool [29]. The protocol of examining the similarity between different samples, evaluating the distances between microbial communities from each sample, and comparing the membership and structure of communities in different samples was followed as described previously [30, 31].

### 2.6. Metabolomics of Liver Tissue Analysis Using the UPLC-Q-TOF/MS Method and Correlation Analysis

The experimental site is in Shanghai Applied Protein Technology Co., Ltd, Shanghai, China. The liver tissue samples were collected in sterile Eppendorf tubes and kept at −80°C until analysis. Pretreatment of the specimen was carried out by operation manual. After that, we used UHPLC (1290 Infinity LC, Agilent Technologies) coupled with a quadrupole time-of-flight (AB Sciex TripleTOF 6600) to perform analyses. After uploading the processed data to MetaboAnalyst (version 4.0, https://www.metaboanalyst.ca) for further analysis, we used principal component analysis (PCA) and partial least squares discriminant analysis (PLS-DA) on the positive and negative models. The final processing of datasets and analysis of results referencing published literature [32]. Then, the correlation analysis between gut microbiota and metabolomics was followed as reported [33]. Differentially abundant gut microbiota and metabolites were log_2_ scaled (TMT/iTRAQ) or Z-score scaled (Label-free) and concatenated into one matrix. Then, correlation coefficient among all the molecules in the matrix was calculated with the Pearson algorithm in R (version 3.5.1, https://www.r-project.org). Pearson correlation coefficient among the differentially expressed gut microbiota and metabolites was loaded into Cytoscape (version 3.5.1, https://www.cytoscape.org) and the correlation network was calculated.

### 2.7. Statistical Analysis

Pooled data are presented as the mean ± SEM. Using SPSS Statistics 24 (IBM, Armonk, New York, United States) for one-way ANOVA followed by Tukey's test to find a significant difference between the tested groups (*P* < 0.05).

## 3. Results

### 3.1. Reduction of HBV Clinical Symptoms by *C. nutans*

**Figure 1:**
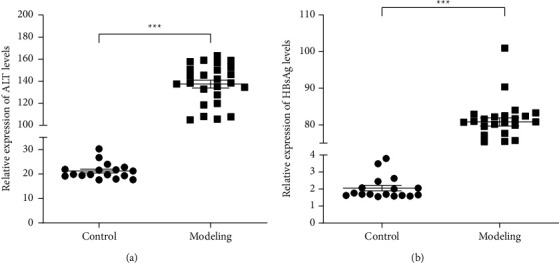
Induction of the HBV mouse model. We detect ALT (a) and HBsAg (b) by ELISA; data represent the mean ± SD (*n* = 20). ^*∗∗∗*^*P* < 0.001 for significant difference of HI HBV plasmid (modeling) compared with the control group.

**Figure 2:**
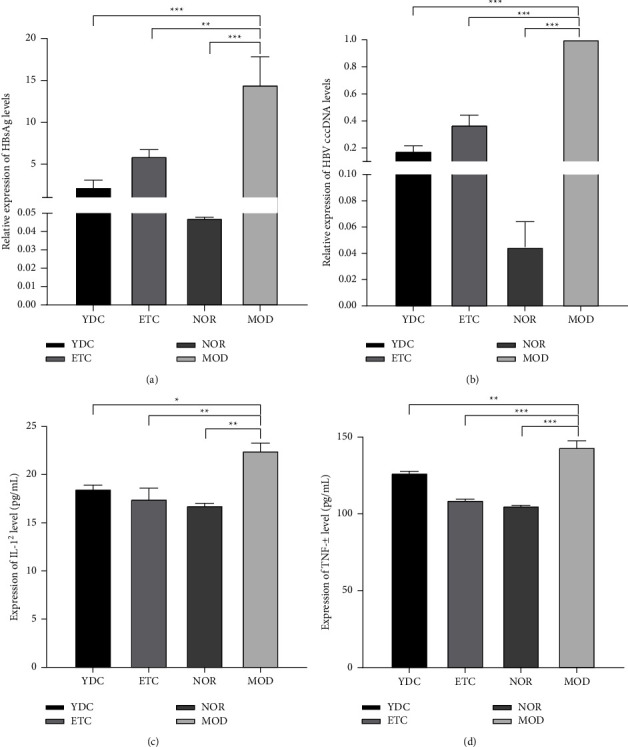
The effect of *C. nutans* on HBsAg, IL-1*β*, TNF-*α*, and cccDNA. We detect HBsAg (a), detect cccDNA (b), and IL-1*β* (c) by ELISA and TNF-*α* (d) by qPCR. Data represent the mean ± SD (*n* = 10). In ELISA, ^*∗*^*P* < 0.05, ^*∗∗*^*P* < 0.01, and^*∗∗∗*^*P* < 0.001 for significant difference of various reagents-treated group compared with the model group. In qPCR, ^*∗∗∗*^*P* < 0.001 for significant difference of relative expression of cccDNA between various reagents-treated group and model group.

We observed a dramatically increasing (*P* < 0.001) in ALT activity and HBsAg levels in serum in the modeling group compared with the control group (see Figures 1(a) and 1(b)). It demonstrated that the HBV model was successfully induced by using HI. To determine whether *C. nutans* had alleviative efficacy on HBV symptoms, HBsAg, IL-1*β*, and TNF-*α* levels in the serum were measured by ELISA, and cccDNA levels in the liver tissues were detected by qPCR, the results show that the *C. nutans* treated group could be observed a significant decline of HBsAg (*P* < 0.001), IL-1*β*(*P* < 0.05), TNF-*α*(*P* < 0.01) levels, and HBV cccDNA (*P* < 0.001) levels compared with the modeling group (see Figures 2(a)–2(d)), showing equivalent or better suppressive effects on HBV than Entecavir.

### 3.2. Changes of the Intestinal Microbial Community by *C. nutans*

**Figure 3:**
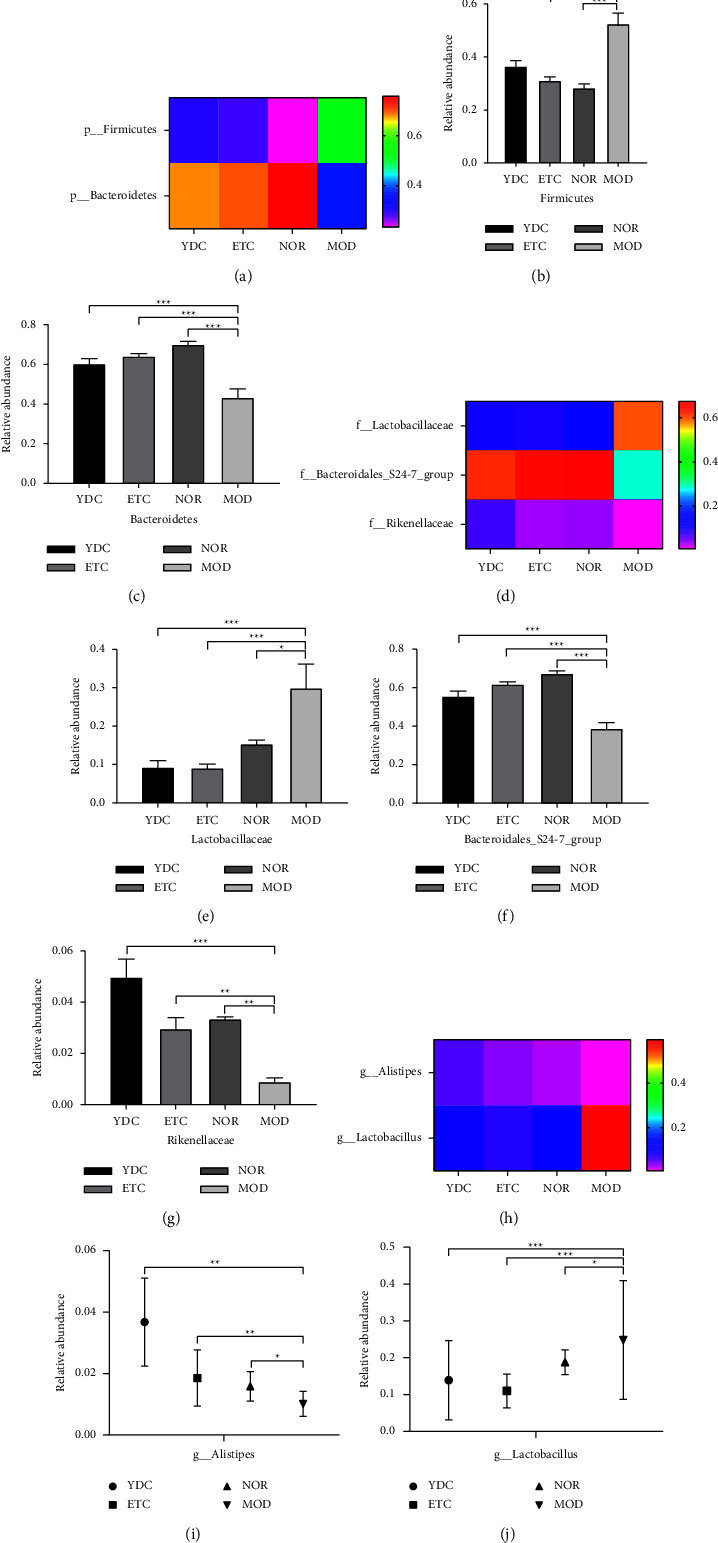
Effects of *C. nutans* on the gut microbial community at phylum, family, and genus level. The heatmap of the microbiota was assessed at the phylum level (a). Data presented are mean ± SEM (*n* = 6); ^*∗∗∗*^*P* < 0.001 for significant difference of relative abundance of Firmicutes (b) and Bacteroidetes (c) between various reagents-treated group and model group. The heatmap of the microbiota was assessed at the family level (d). Data are presented mean ± SEM (*n* = 6); ^*∗*^*P* < 0.05, ^*∗∗*^*P* < 0.01, and^*∗∗∗*^*P* < 0.001 for significant difference of relative abundance of *Lactobacillaceae* (e), *Bacteroidales_S24-7_group* (f), and *Rikenellaceae* (g) between various reagents-treated group and model group. The heatmap of the microbiota was assessed at the genus level (h) and the difference of relative abundance of various bacteria (i, j) between various reagents-treated group and model group.

We performed the PCA method, which is based on the relative abundance of genera, to obtain an overview of the microbiota composition between the modeling group mice and the nondisease group, and the results revealed that the group of YDC and ETC, separated from the model group mice (see Figure S1a), showed a significant difference in gut microbiota in each group. The gut microbiota diversity of the mice is shown in Figure S1c–S1f, the results show that the diversity index of Shannon, chao1, observed_species, and PD_whole_tree was higher in the NOR, YDC, and ETC groups than that in the MOD group. Specific gut microbiota changes in MOD and YDC group mice were accessed across levels of phylum, family, and genus. The MOD group mice had a significant increase of Firmicutes (*P* < 0.001) and a decrease of Bacteroidetes (*P* < 0.001) compared with the NOR group mice at the phylum level (see Figure 3(a)). Gut microbiota communities of the mice in the group of YDC and ETC showed a trend of increase of Bacteriodetes (*P* < 0.001) and decrease of Firmicutes (*P* < 0.001), compared with those of the MOD group mice (see Figures 3(b) and 3(c)). At the family level, the MOD group mice had a dramatic reduction of *Bacteroidales_S24-7_group*(*P* < 0.001) and *Rikenellaceae*(*P* < 0.01), showing a very significant increase of *Lactobacillaceae*(*P* < 0.05) compared with NOR group mice (see Figure 3(d)). Gut microbiota communities of the mice in the group of YDC and ETC showed a trend of increase of *Bacteroidales_S24-7_group*, *Rikenellaceae* and decline of *Lactobacillaceae* (see Figures 3(e)–3(g)). At the genus level, the group of MOD mice had a dramatic decrease of *Alistipes*(*P* < 0.05) but a significant increase of *Lactobacillus*(*P* < 0.05), compared with the NOR group (see Figure 3(h)). The *C. nutans* and Entecavir treatment led to a sharp decrease in *Lactobacillus*(*P* < 0.001) and led to an increase in *Alistipes*(*P* < 0.01) (see Figures 3(i) and 3(j)). These results show that the gut microbiota in HBV-model mice was modulated by *C. nutans*.

### 3.3. Changes of Liver Tissue Metabolites by *C. nutans*

Comparing the total ion flow diagram of the QC sample UHPLC-Q-TOF MS with the overlapping spectrograms, the results in Figure S2 show that the response intensity and retention time of each color spectrum peak basically overlap, indicating that the variation caused by the instrument error is small in the whole experiment process. OPLS-DA, based on the relative abundance of positive and negative ion metabolites, revealed the model group mice showed a distinct microbiota composition from the other groups (see Figures S3a and S3b). The volcano figures show the differential metabolites of positive and negative ions between the group of YDC and MOD, and the significant differential metabolites were in pink color (see Figures S2c and S2d). We applied the VIP value >1 and the *P* < 0.05 analysis to Student's *t*-test at a univariate level to measure the significance of each metabolite and screen 9 common upregulated differential metabolites and 14 common downregulated differential metabolites (see Table 1). The results showed that *C*. *nutans* dramatically upregulate hippuric acid, L-histidine, trehalose, D-threitol, and stachyose and downregulate uridine 5'-diphosphate, cholic acid, trimethylamine N-oxide, CDP-ethanolamine, and phosphorylcholine (*P* < 0.05).

### 3.4. Changes of the Intestinal Microbial Community and Influence of Liver Tissue Metabolites by C. nutans

**Figure 4:**
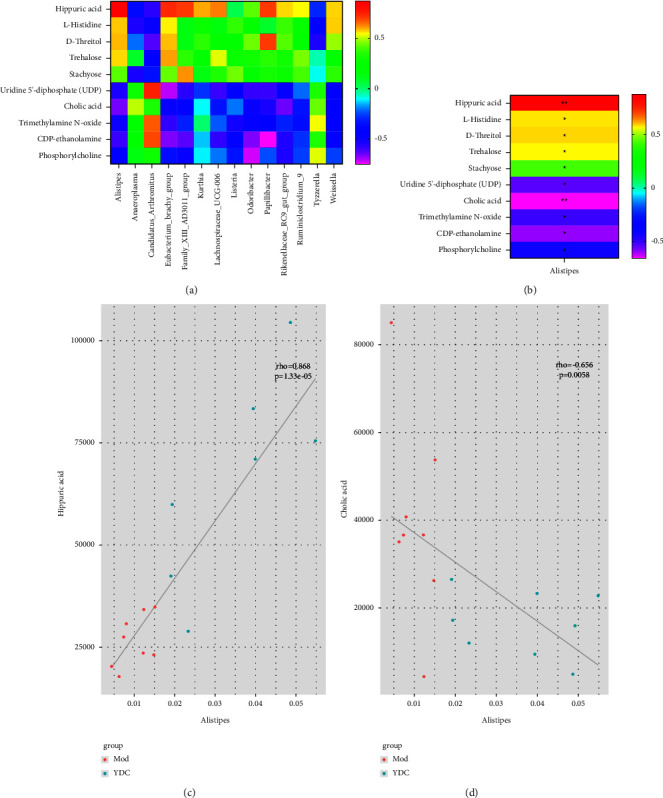
Correlation analysis of differentially abundant gut microbiota and metabolites. The Spearman's correlation heatmap of differentially abundant gut microbiota and metabolites (a); the column indicates the genus of bacteria, the row indicates the metabolite, the correlation coefficient R is expressed in color, and the deeper the color, the stronger the correlation. The correlation heatmap between *Alistipes* and differentially metabolites (b); *P* value reflects the significant level of correlation; 0.01 < *P* value <0.05, expressed as ^*∗*^; *P* value <0.01, expressed as ^*∗∗*^. The Spearman correlation between *Alistipes* and associated metabolites (c, d), the rho shown in the upper left corner of each figure is the correlation coefficient between the relative abundance of the strain and the metabolite intensity value, and the *P* value is the significant level of the rho ((c) rho = 0.868, *P* = 1.33e-5; (d) rho = −0.656, *P* = 0.0058). The red dots are the samples of the MOD, and the green dots are the samples of the YDC.

From the results in Figure 4(a), *Alistipes* has a high correlation with the significantly different metabolites screened out, and it can be seen from the color that *Alistipes* are positively correlated with upregulated metabolites, such as hippuric acid, L-histidine, trehalose, D-threitol, and stachyose and showed negativity related with downregulated metabolites, such as uridine 5′-diphosphate, cholic acid, trimethylamine N-oxide, CDP-ethanolamine, and phosphorylcholine. In Figure 4(b), *Alistipes* is the genus with the most significant correlation of the significantly different metabolites screened, showing a significant negative correlation with uridine 5′-diphosphate, trimethylamine oxide, CDP ethanolamine and phosphorylcholine (*P* < 0.05), a significant negative correlation with cholic acid (*P* < 0.01) a significant positive correlation with hippuric acid (*P* < 0.01), a significant positive correlation with L-histidine, trehalose, D-threitol, and stachyose (*P* < 0.05). In Figure S4, *Alistipes* is in the most prominent position, suggesting that it is a crucial intestinal microbe. In Figures 4(c) and 4(d), the degree of dispersion shows the distribution of *Alistipes* and differential metabolites in the two samples. The metabolite rho value of *Alistipes* positive regulation is hippuric acid (Rho = 0.868, *P* = 1.33e-5, see Figure 4(c)). The negatively regulated metabolite rho has the largest absolute value for cholic acid (rho = −0.656, *P* = 0.0058, see Figure 4(d)).

## 4. Discussion

*C. nutans*, as a traditional herb, shows anti-inflammatory activity, immunomodulating activity, and antiviral activity [34], but its effect on HBV has rarely been reported. In this paper, we observed its anti-HBV infection efficacy. *C. nutans* directly interfered with the production of HBsAg, IL-1*β*, TNF-*α* in serum, and HBV cccDNA in the liver after analyzing all the experimental data. *C*. *nutans* also shows the impact of changing the gut microbial community and liver metabolites in HBV model mice. In addition, from the monitoring results of the body weight and health conditions of mice in all groups, *C. nutans* is a biosafety and low-cost green medicinal plant that can be used for the long-term use.

*C. nutans* has noticeable efficacy in alleviating symptoms of HBV infection. Previous studies demonstrated that continued high levels of HBsAg are accompanied by low immune function in chronic hepatitis B (CHB) patients, and the long-term high titer of HBsAg may cause the degree and function of HBV-specific immune cells to become resistant [35, 36]. In this work, *C. nutans* can significantly reduce the serum HBsAg level of model mice, showing the potential to alleviate the failure of immune cell function and carry out immune regulation. In addition, it is reported that elevated levels of inflammatory cytokines, such as TNF-*α* and IL-1*β*, could be observed in the serum of HBV-infected persons, promoting liver inflammation and malignant progression [37–39]. As displayed in our results, *C. nutans* significantly reduced the serum levels of TNF-*α* and IL-1*β* in model mice and showed an excellent anti-inflammatory effect and indicated that the efficacy of *C. nutans* on HB progression could partly be attributed to its ability to inhibit the secretion of inflammatory cytokines. Moreover, it is necessary to eliminate the nuclear cccDNA from the infected liver cells for long-term antiviral therapy [40, 41]. In the present study, *C. nutans* can significantly inhibit the level of cccDNA in mouse liver cell nuclear and exhibits excellent antiviral performance and long-term efficacy. However, further research and more comprehensive data are needed to reveal the mechanism that has not been clarified yet. In short, our results proved that *C. nutans* exhibits significant efficacy in inhibiting HBV infection and HB progression.

Investigating the gut microbiota may provide a new perspective into the origination and development of HB. As demonstrated in the past research, the imbalance of the gut microbiota is closely related to the development of CHB disease [8]. After acute or chronic HBV infection in mice, the richness of the Bacteroidetes phyla significantly declined. In contrast, the richness of Firmicutes dramatically increased considerably, indicating that the ratio of Firmicutes/Bacteroidetes (F/B) significantly increased dramatically after HBV infection [42]. In our work, the modeling mice had a significant increase of Firmicutes and a decrease of Bacteroidetes, further confirming the successful establishment of the HBV mouse model from another aspect. Gut microbiota communities of the mice in *C. nutans* and Entecavir treated groups showed a trend of increase of Bacteroidetes and a decrease of Firmicutes, indicating that *C. nutans* may intervene in the progression of HB by changing the ratio of F/B at the phylum level of gut microbiota, as same as Entecavir do.

In addition, *C. nutans* can significantly reduce the relative abundance of *Lactobacillaceae* at the family level and *Lactobacillus* at the genus level, shows obvious inhibitory regulation on *Lactobacillus*-ssp. As reported in the literature that *Lactobacillus*-ssp has obvious pathogenicity and can cause endocarditis and meningitis [43]. In addition, *Lactobacillus paracasei* and *Lactobacillus rhamnosus* isolated from the drainage tube of patients are closely correlated with infections, and *Lactobacillus gasseri* can be both pathogenic and colonizing bacteria [44]. Although the role of these bacteria in the progression of HB has not been confirmed, it provides a guide for us to further explore the effect of *C. nutans* on intestinal flora against infectious diseases including HB. Moreover, compared with the modeling group, *C. nutans* can significantly increase the abundance of *Rikenellaceae* at the family level and *Alistipes* at the genus level in the intestines of mice. *Alistipes* belongs to the Bacteroidetes phyla and *Rikenellaceae* family, indicating that *C. nutans* may directly act on increasing the quantity of *Alistipes* to achieve the upregulation of Bacteroidetes and finally restore the ratio imbalance of F/B.

Metabolomics research provides a new weapon for us to evaluate the body's condition from disease to recovery. As shown in our results, the upregulated differential metabolites shared by the group of YDC and NOR and/or ETC include hippuric acid and L-histidine. These metabolites may be the target metabolites of *C. nutans* to restore the original level of metabolites in the body. The unique upregulated metabolites of the YDC group are mainly trehalose, D-threitol, and stachyose. These metabolites may have the ability in the transformation of the practical components of the grass. Furthermore, the downregulation of the differential metabolites, shared by the group of YDC and NOR and/or ETC, including uridine 5′-diphosphate, cholic acid, trimethylamine oxide, CDP ethanolamine, and phosphorylcholine. The above data confirm that *C. nutans* exhibits the potential of leading the decrease of oxidative stress in the body and the production of TNF-*α* in the liver by inhibiting the level of these harmful metabolites [45].

The correlation analysis between gut microbiota and liver metabolomics helps us screen out the target-regulated bacteria and the critically related metabolites by *C. nutans* on HB. As displayed in Figure S4 and Figure 4, *Alistipes* is a crucial intestinal microbe, which is consistent with the results of intestinal flora screening. The results of Figures 4(c) and 4(d) show that the positive regulation metabolite of *Alistipes* is hippuric acid while the negatively regulated one is cholic acid, agrees with the results of the moderation trends both in different gut microbiota and in different liver metabolites. The published literature revealed that in healthy people, the abundance of *Alistipes* is significantly higher than in HBV-infected patients [46]. When HBV infection progresses to cause a more severe disease stage, it is reduced dramatically, other studies have also shown that CHB patients had a significant reduction in *Alistipes* compared to healthy patients [47], which implies the potential benefits of *Alistipes*. Hippuric acid is one of the metabolites derived from intestinal microbes, which can be used as an indicator of microbial diversity, and its derivatives have anti-inflammatory and anti-HBV activity [48, 49]. Cholic acid are considered an interventive targets for relieving liver inflammation as well as a potential biomarker for targeted therapy of HBV-infected patients [50–52]. All the results imply that the content of *Alistipes* is increased through the regulation of *C. nutans* and then directly affects the metabolism levels of hippuric acid and cholic acid, which is the key potential metabolic pathway for the efficacy of *C. nutans* on HB.

## 5. Conclusion

In conclusion, *C. nutans* is a reliable drug for the prevention and treatment of HB, it exerts a drug effect that alleviative the HBV symptoms in mouse models and has remodeled gut microbiota in mice and modulate the level of liver metabolites to prevent and treat HB. *C. nutans* shows ability in regulating the crucial bacteria *Alistipes*, and then restrict the metabolism levels of hippuric acid and cholic acid to play a specific role in preventing and curing HB.

## Figures and Tables

**Table 1:** Differential metabolites.

Description	Fold change	VIP	*P* value	Label
D-maltose	3.812	2.086	0.0062	POS
L-histidine	2.915	4.019	0.0238	POS
Hippuric acid	2.529	1.563	0.0003	POS
Stachyose	2.413	1.545	0.0028	POS
Maltotriose	2.4065	1.035	0.0103	POS
D-threitol	1.466	1.268	0.0161	POS
Trehalose	1.326	1.131	0.0195	POS
D-mannose	1.236	1.772	0.0086	POS
Pantothenate	1.224	3.251	0.0148	POS
Hypoxanthine	0.840	9.734	0.0257	NEG
L-phenylalanine	0.829	4.627	0.0105	NEG
L-glutamate	0.792	1.024	0.0336	NEG
Tyramine	0.791	3.616	0.0028	NEG
Phosphorylcholine	0.782	2.272	0.0305	NEG
L-threonate	0.749	5.935	0.0088	NEG
Trimethylamine N-oxide	0.669	1.885	0.0006	NEG
Cytidine 5′-diphosphocholine	0.598	2.470	0.0027	NEG
Homocitrate	0.594	1.028	9.9E-05	NEG
Phosphorylcholine	0.571	1.713	0.0335	NEG
Hypoxanthine	0.555	4.512	0.0044	NEG
CDP-ethanolamine	0.545	1.111	0.0008	NEG
Cholic acid	0.414	1.098	0.0167	NEG
Uridine 5′-diphosphate (UDP)	0.166	1.522	0.0055	NEG

Metabolites with the VIP value >1 was further applied to Student's *t*-test at univariate level to measure the significance of each metabolite; *P* < 0.05 were considered as statistically significant. The label POS represents upregulated metabolites, and NEG represents downregulated metabolites.

## Data Availability

The data used to support the findings of this study are available from the corresponding authors upon request.

## Supplementary Materials

Supplementary Materials This supplementary material included all the research results mentioned in the manuscript. Figure S1: effects of *C. nutans* for the HBV mouse model on the gut microbial community. Figure S2: positive/negative ion mode TIC overlapping Atlas of samples. Figure S3: effects of *C. nutans* for the HBV mouse model on the liver tissue metabolites. Figure S4: degree value analysis of very important bacteria.
