# Supplementary material for: Protective Effects of Clinacanthus nutans (Burm.f.) Lindau Aqueous Extract on HBV Mouse Model by Modulating Gut Microbiota and Liver Metabolomics
Source: Evid Based Complement Alternat Med. 2023 Jan 3;2023:5625222. doi: 10.1155/2023/5625222 (PMC9831714; doi:10.1155/2023/5625222)
Supplement: Supplementary Materials — This supplementary material included all the research results mentioned in the manuscript. Figure S1: effects of C. nutans for the HBV mouse model on the gut microbial community. Figure S2: positive/negative ion mode TIC overlapping Atlas of samples. Figure S3: effects of C. nutans for the HBV mouse model on the liver tissue metabolites. Figure S4: degree value analysis of very important bacteria. [file 5625222.f1.doc]

**Supplemental Files**

**Figure S1. Effects of *C.nutans* for HBV mouse model on the gut microbial community.** The phylogenetic differences within the fecal microbiota were assessed by PCA assay (a). Microbial community structure, richness, and evenness were calculated by Shannon’s richness index (b, f), CHAO1 assay (c), Observed_species assay (d), and PD_whole_tree assay (e).

**Figure S2. Positive/Negative ion mode TIC overlapping atlas of sample.** Positive/Negative ion mode TIC overlapping atlas of QC sample (a, b). Positive/Negative ion mode TIC overlapping atlas of group_YDC sample (c, d). Positive/Negative ion mode TIC overlapping atlas of group_ETC sample (e, f). Positive/Negative ion mode TIC overlapping atlas of group_Nor sample (g, h). Positive/Negative ion mode TIC overlapping atlas of group_Mod sample (i, j).

**Figure S3. Effects of *C.nutans* for HBV mouse model on the liver tissue metabolites.** The up and down-regulation differences within the metabolites were assessed by PCA assay (a) and OPLS-DA assay (b). Differences within the metabolites between *C.nutans* treated group and the model group was shown in the volcano plot (c, d). Metabolites with a VIP value >1 was further applied to Student’s t-test at a univariate level to measure the significance of each metabolite, the *P* values less than 0.05 were considered statistically significant.

**Figure S4. A degree value analysis of very important bacteria.** The circles represent the genus of bacteria and the rectangles represent metabolites. The blue line represents the negative correlation, the red represents the positive correlation, and the thickness of the line shows the correlation coefficient. The node size is positively related to its degree, that is, the greater the degree, the larger the node size.


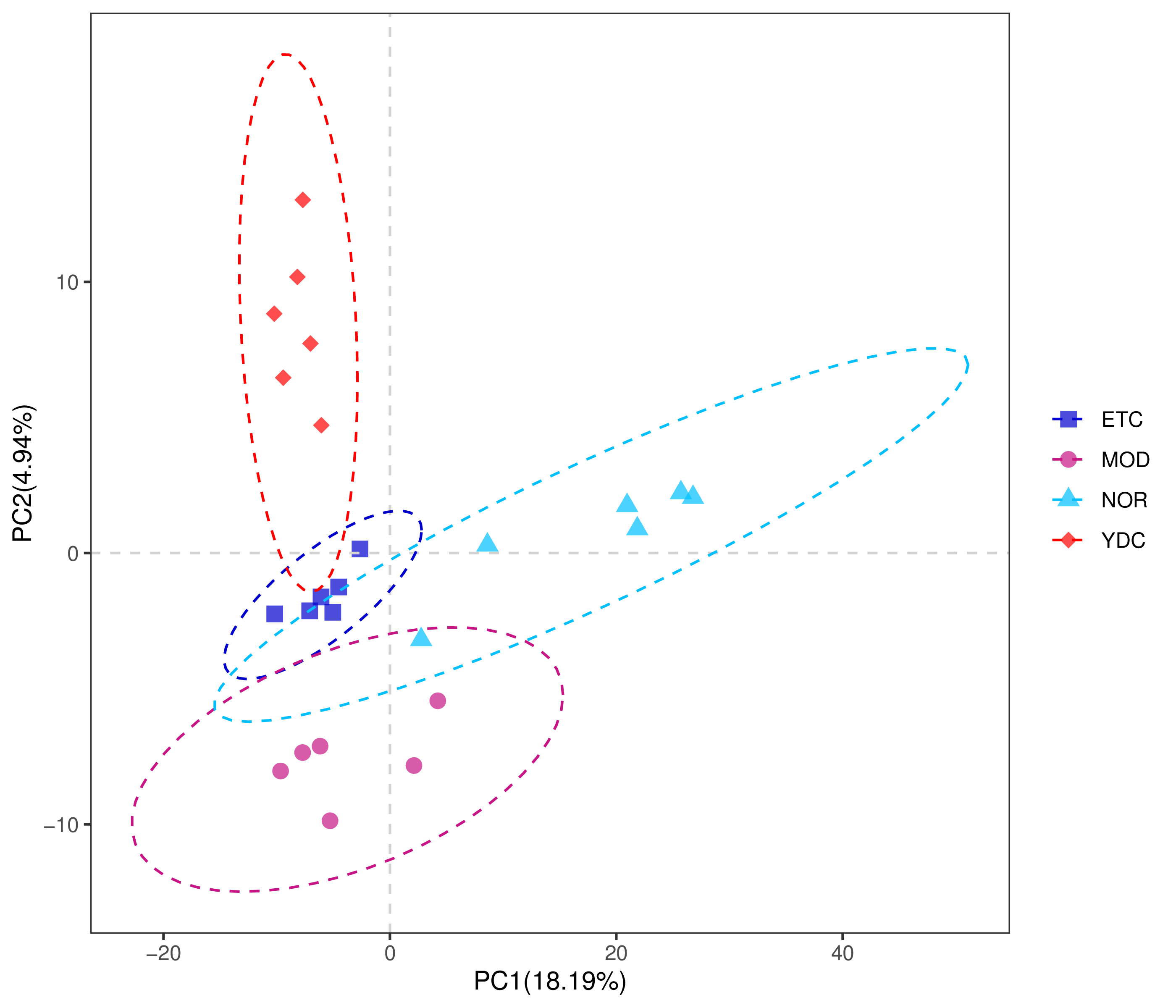

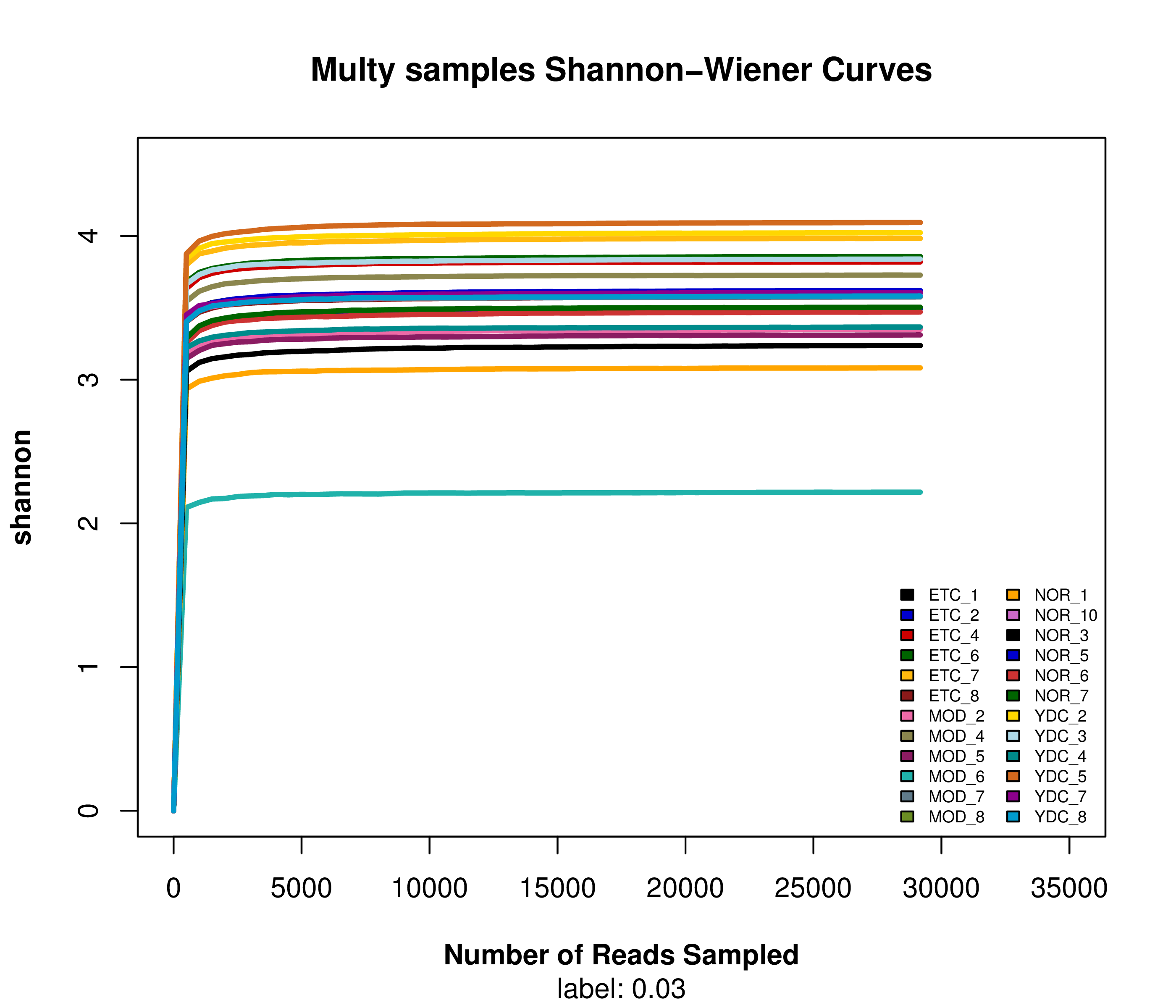
**Fig.S1**


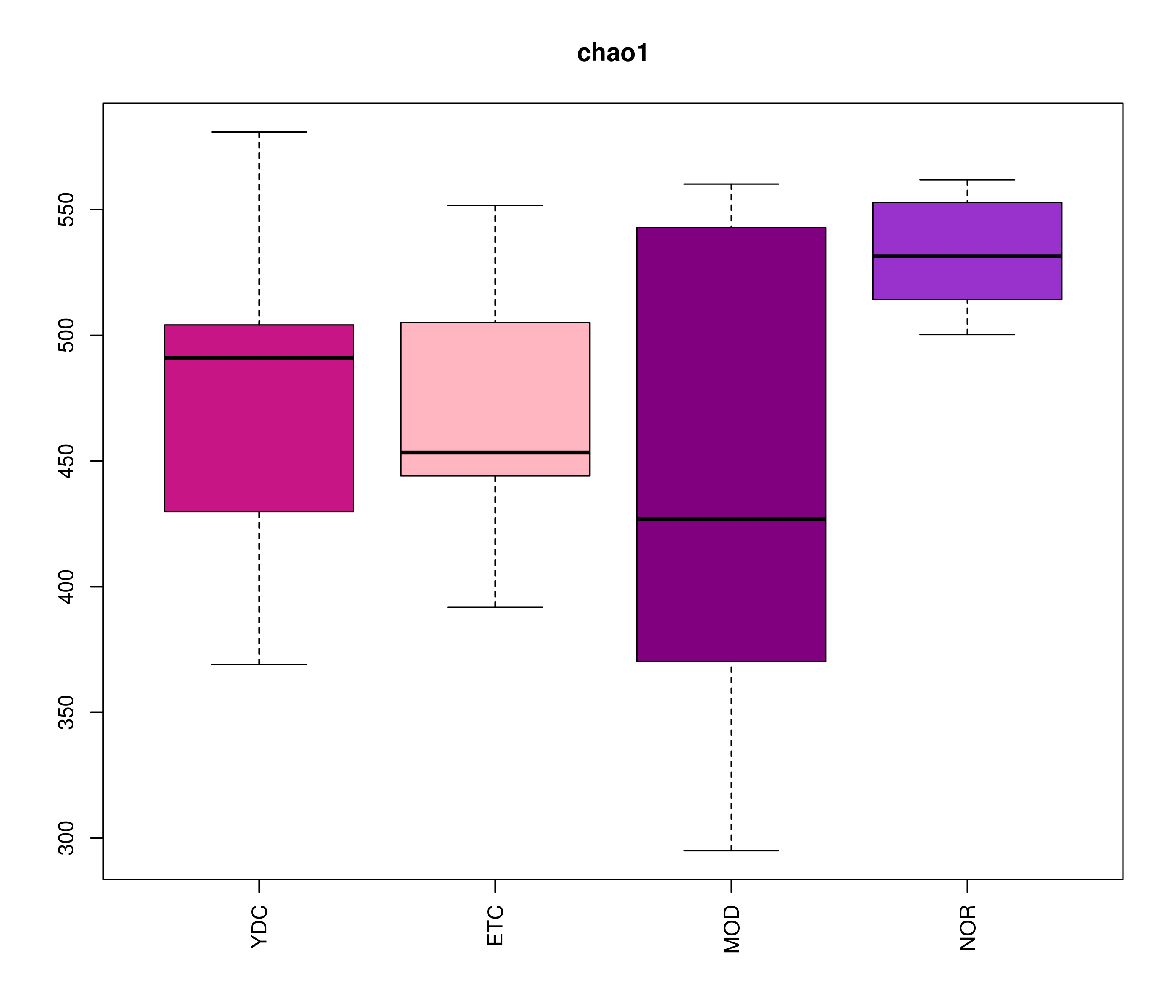

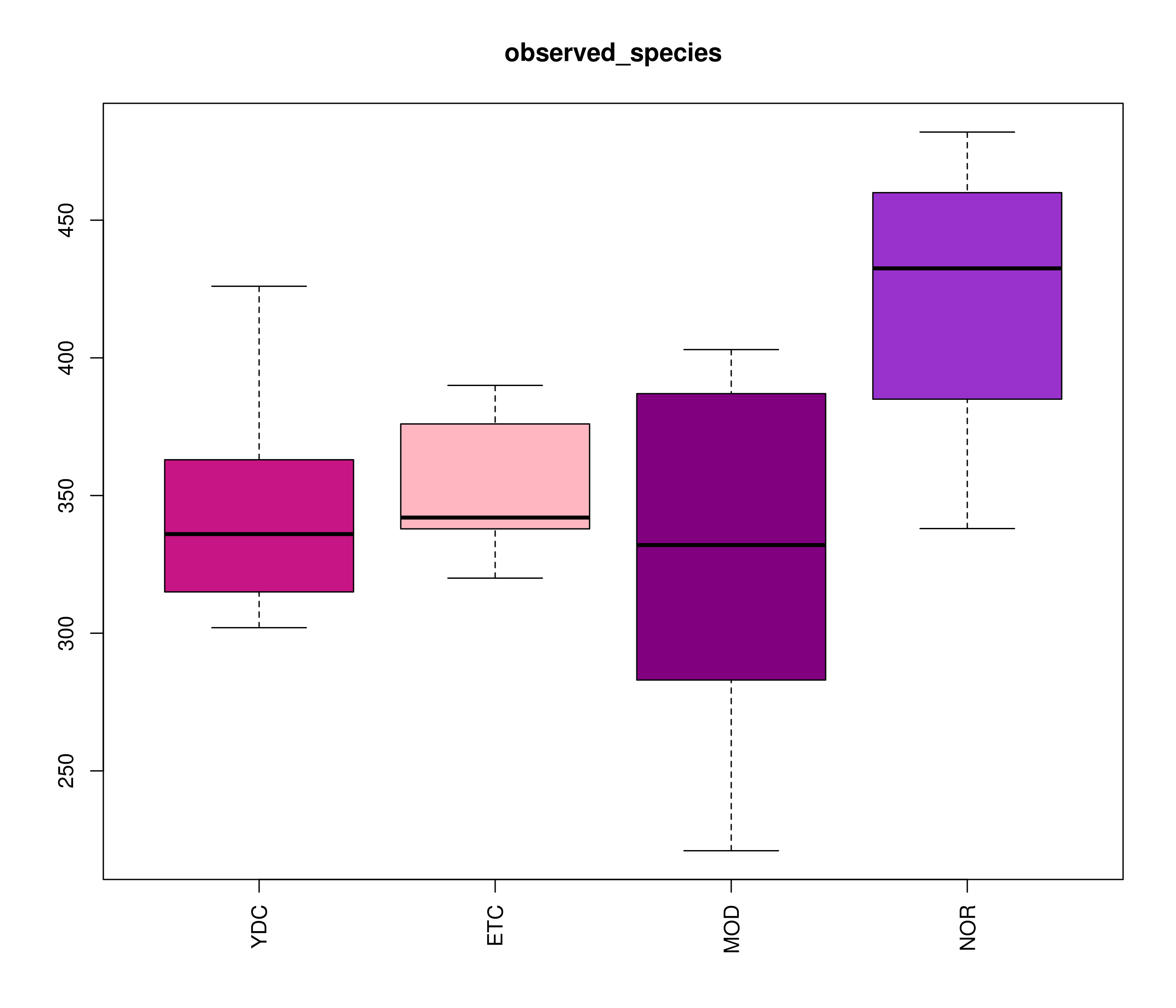
(a) (b)

(c) (d)


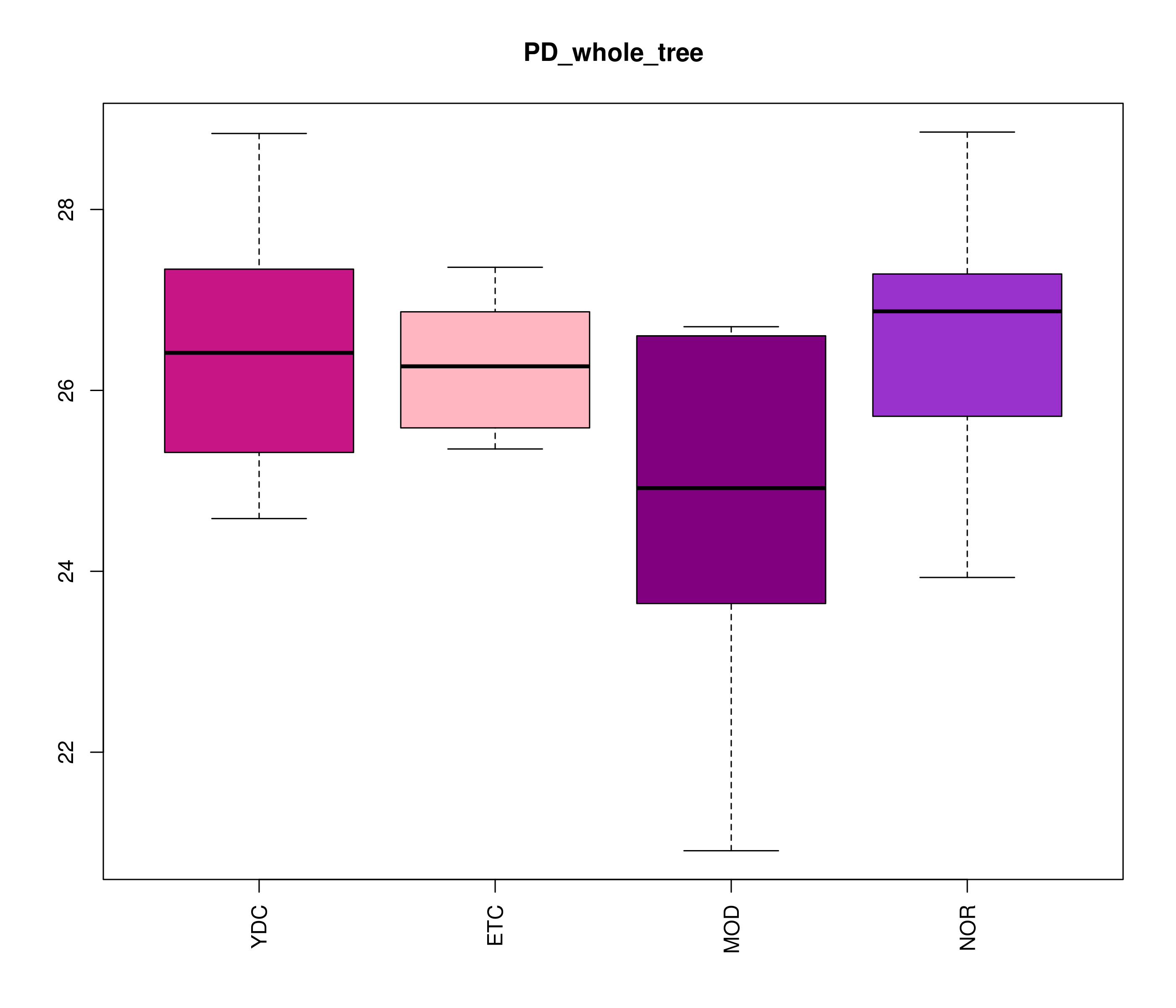

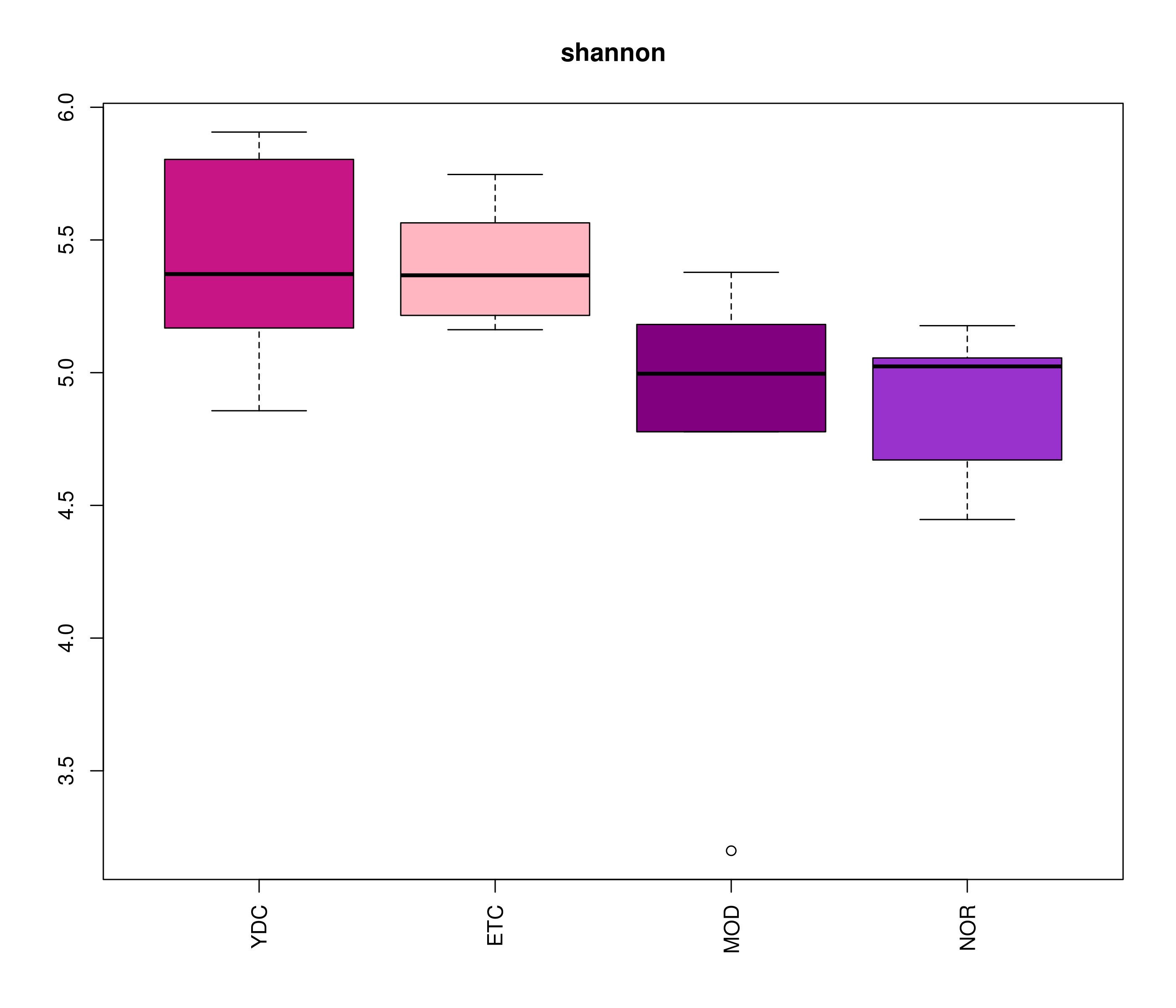


(e) (f)

**Fig.S2**

(a)

(b)

(c)

(d)

(e)

(f)

(g)

(h)

(i)

(j)


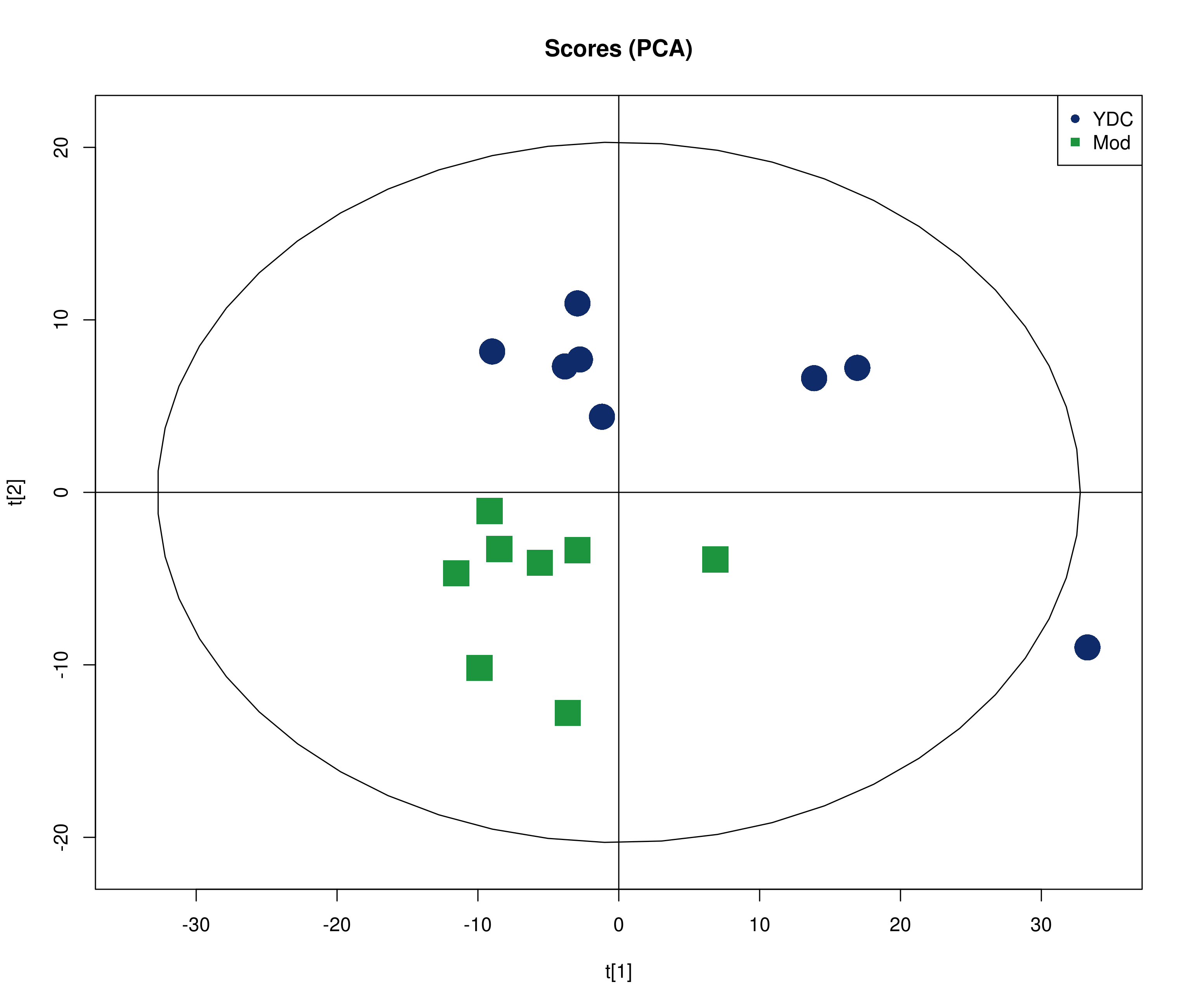

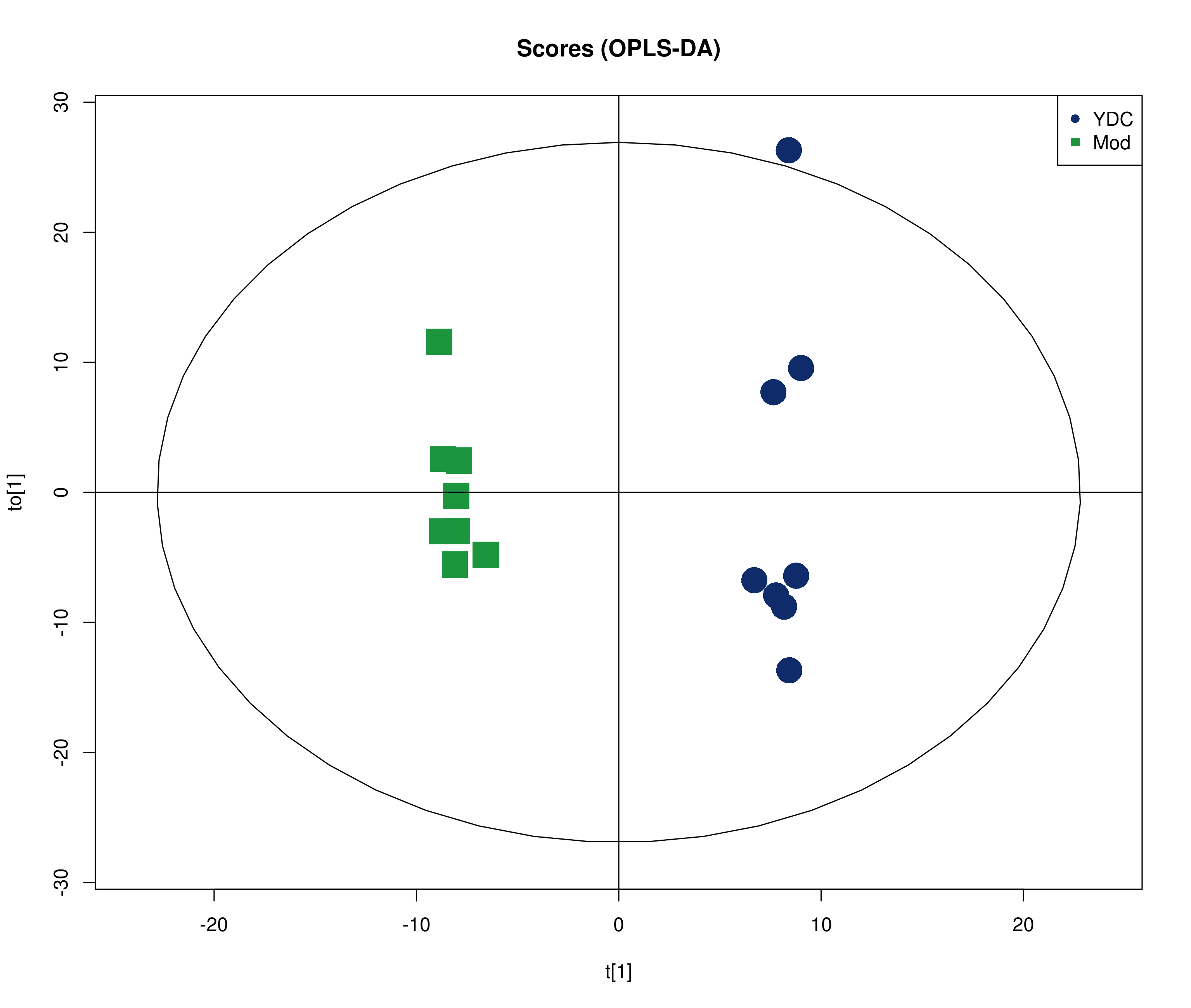
**Fig.S3**

(a)


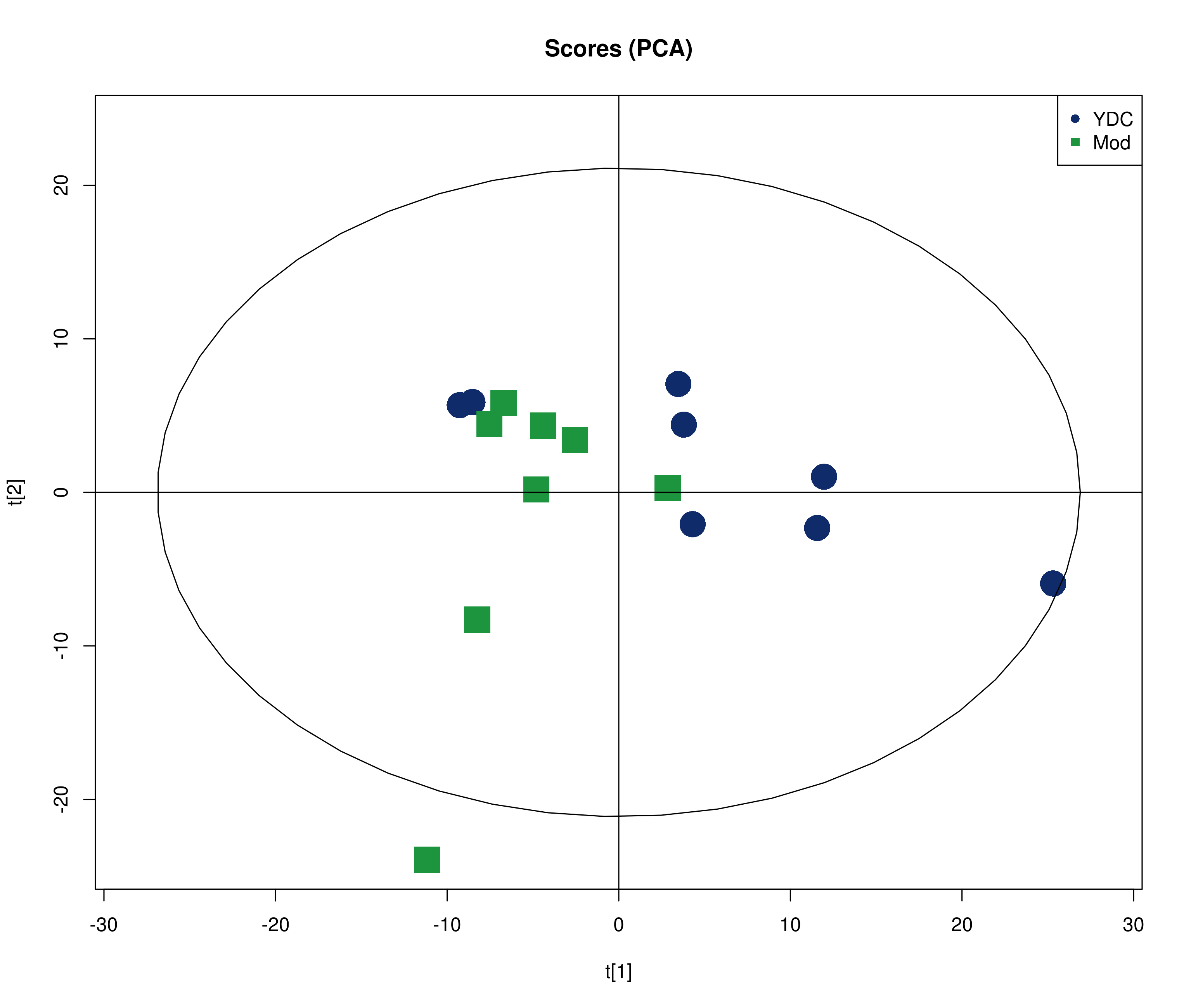

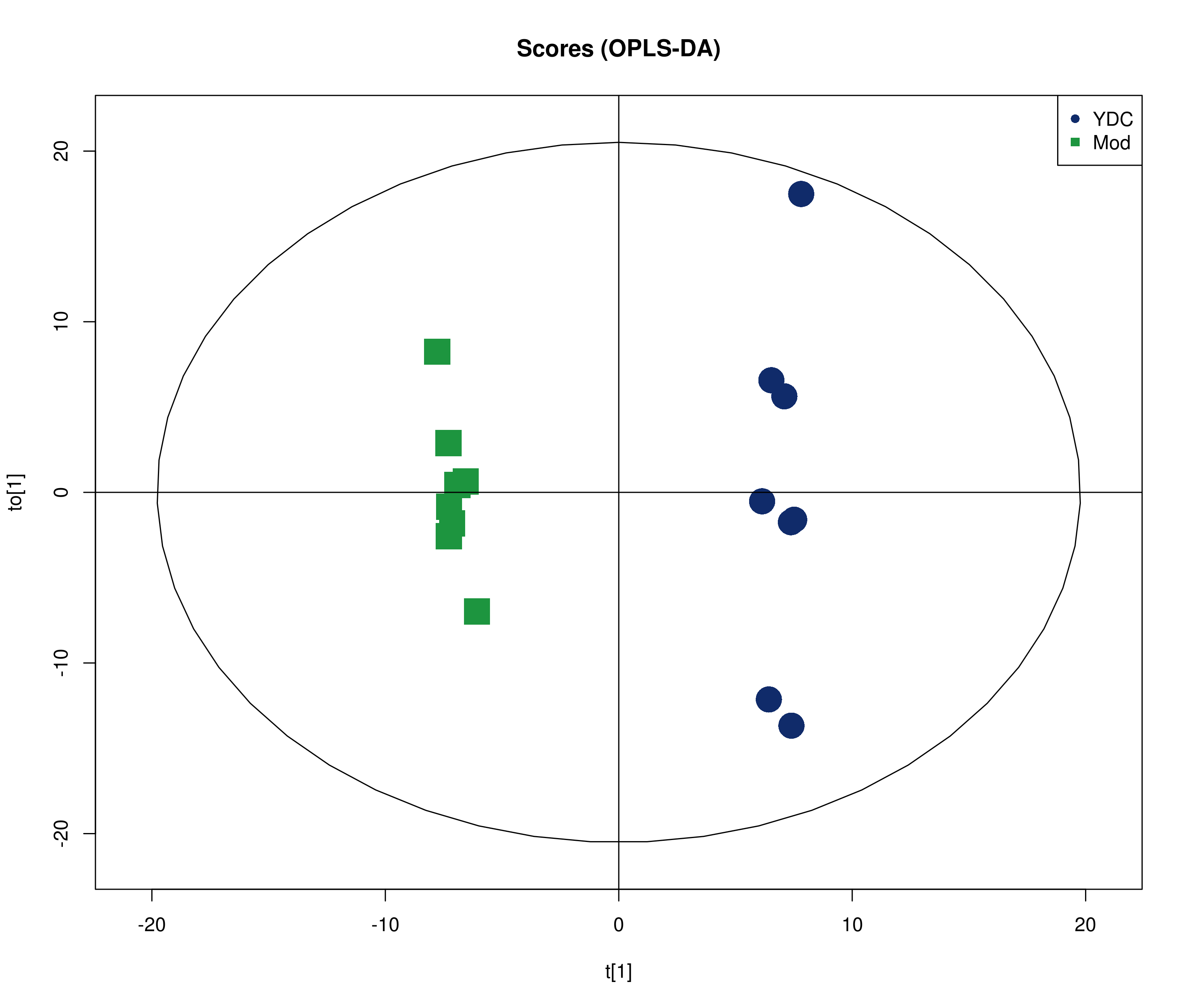


(b)

**
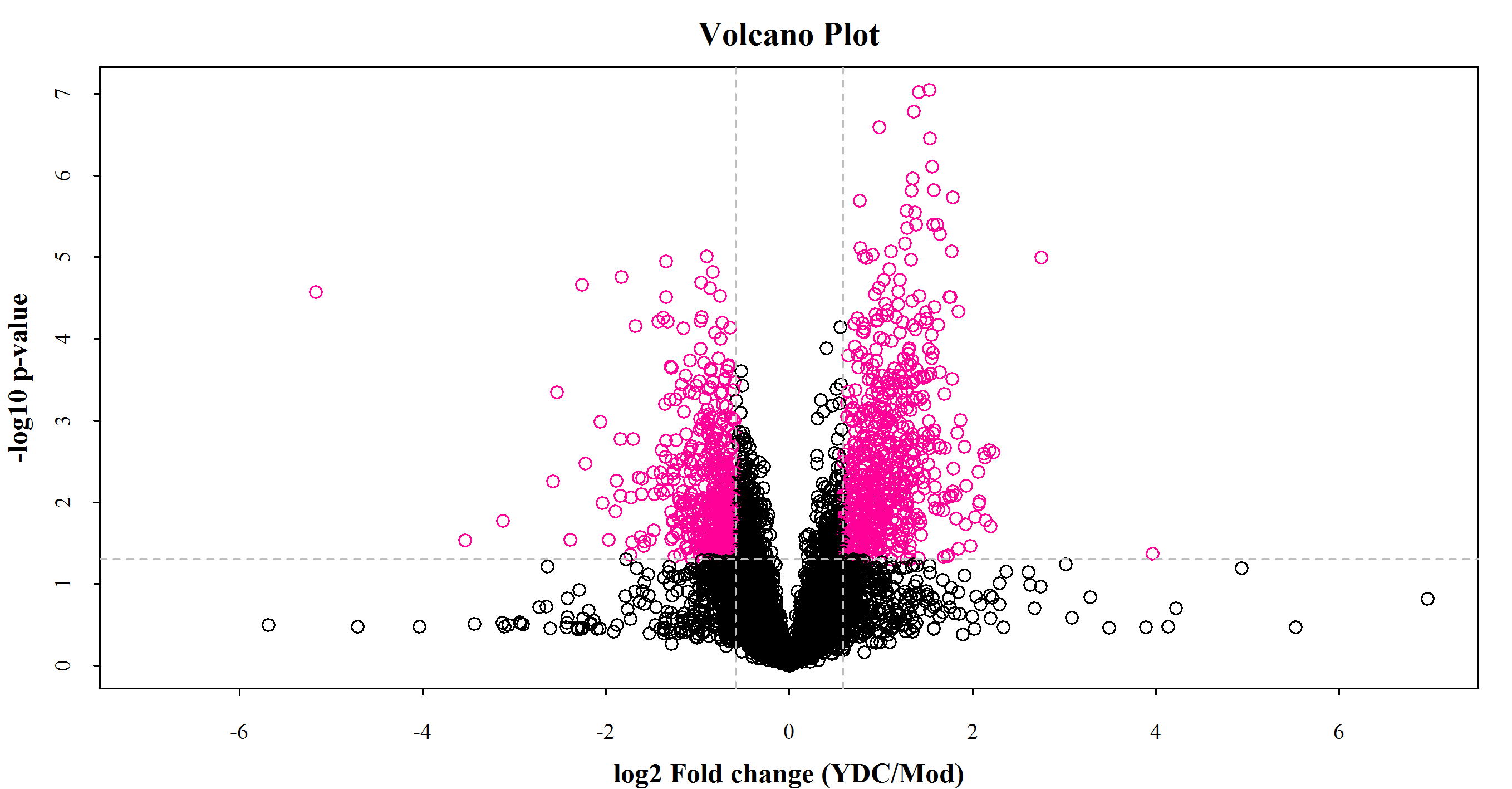
**

(c)

**
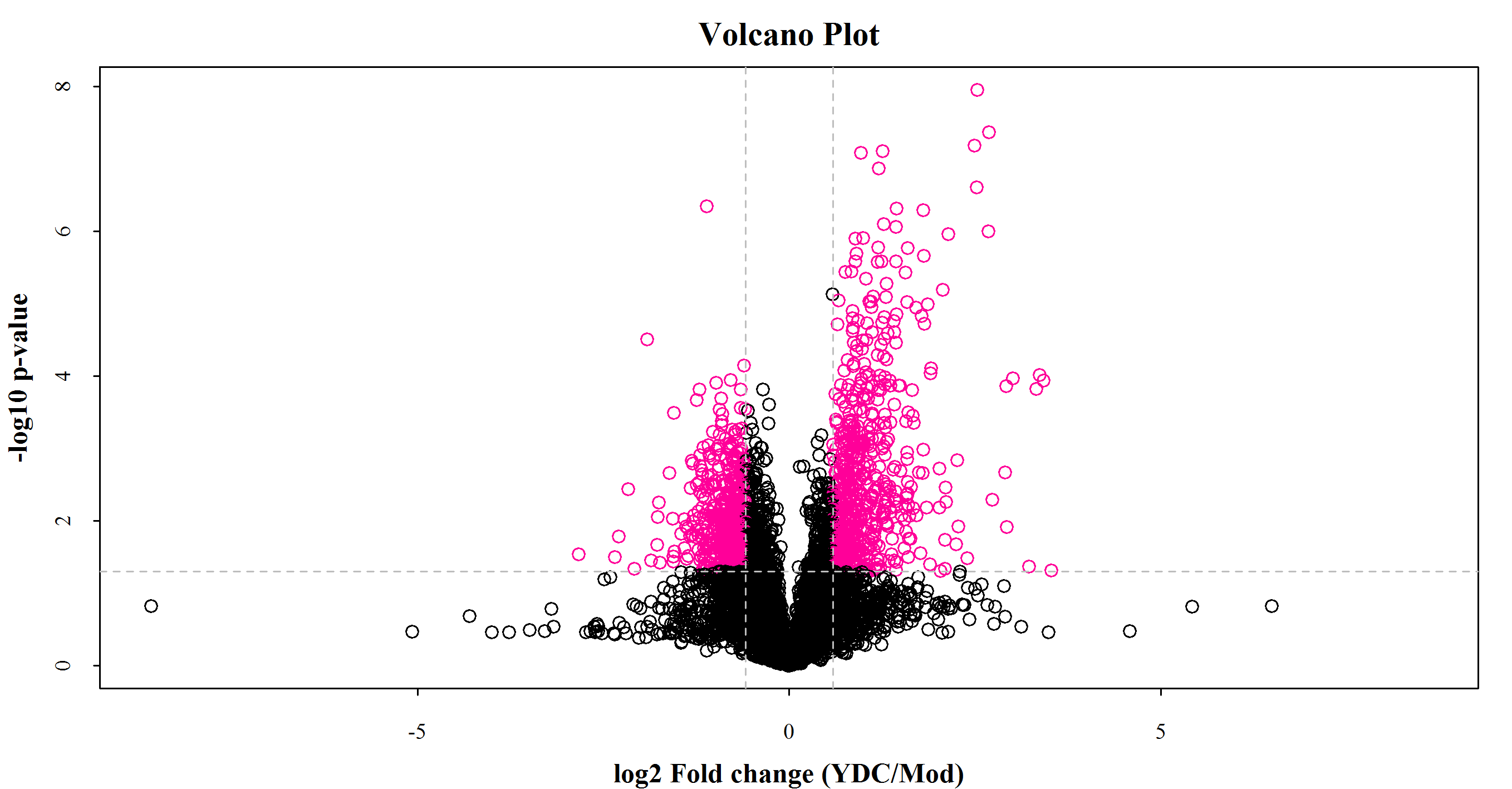
**

(d)

**Fig.S4**

**
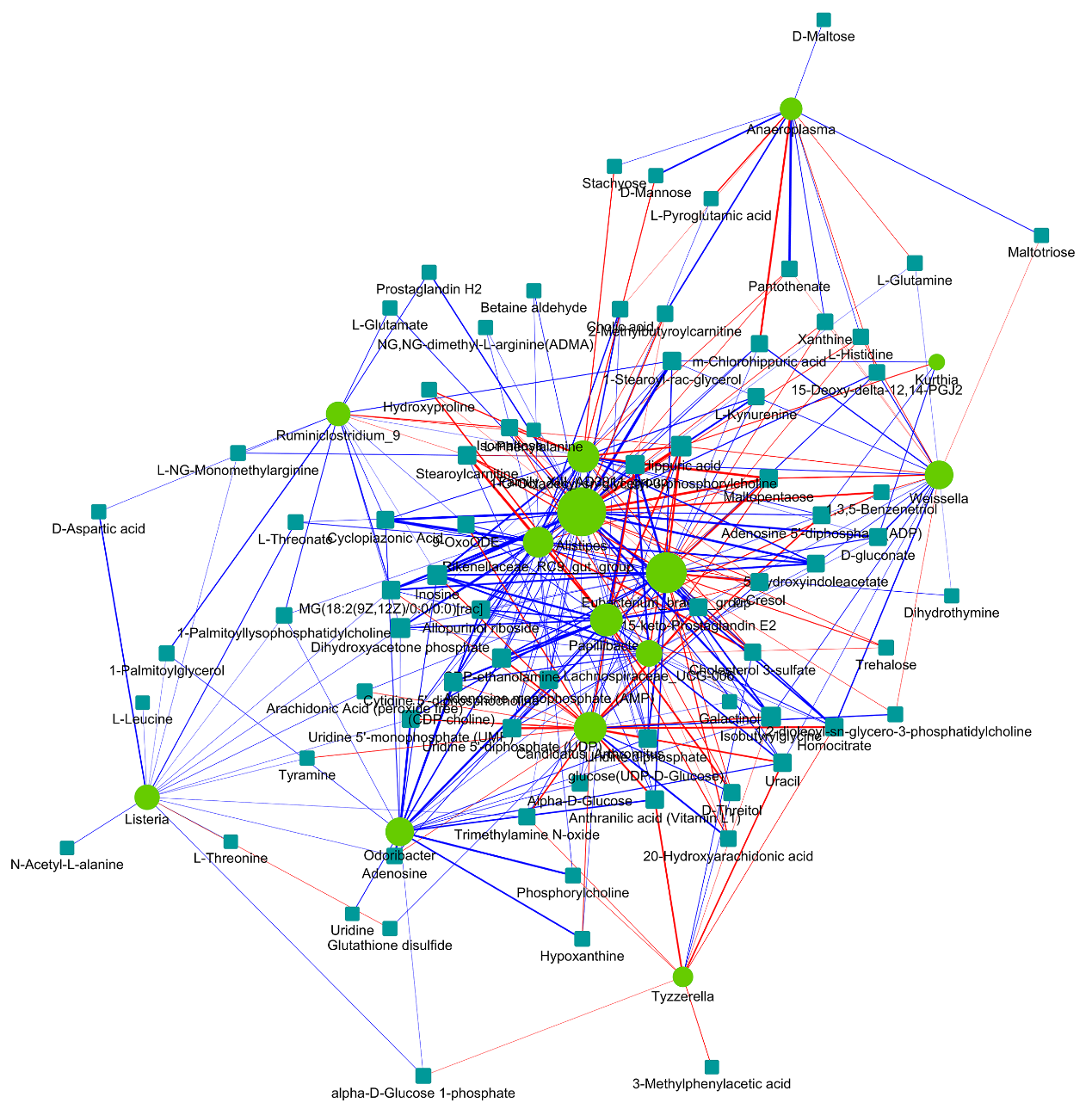
**
